# A new species of *Metacyclops* from a hyporheic habitat in North Vietnam (Crustacea, Copepoda, Cyclopidae)

**DOI:** 10.3897/zookeys.522.5989

**Published:** 2015-09-23

**Authors:** Andrzej Kołaczyński

**Affiliations:** 1 Museum and Institute of Zoology Polish Academy of Sciences, Wilcza 64, 00-679 Warszawa, Poland

**Keywords:** East Asia, freshwater, North Vietnam Cyclopinae, taxonomy, zoogeography

## Abstract

A new species of *Metacyclops* is described from hyporheic waters and small rock depression with leaf litter in North Vietnam, the Tam Đao Mountains). *Metacyclops amicitiae* **sp. n.** can be distinguished from its congeners by the unique combination of the following characters: 12-segmented antennule, distal segment of P4 endopodite bearing a single apical spine, and the surface ornamentation of the intercoxal sclerites in P1–P4 (pilose on the distal margin of P1-P4 and spinulose on the caudal surface of P4). The latter character separates the new *Metacyclops* from its closest relative, *Metacyclops ryukyuensis*, known only from the Ryukyu Islands (Ishigaki). The genus *Metacyclops* with the new species described herein is also for the first time recorded from Vietnam. An identification key is provided to the south and east Asian species of the genus.

## Introduction

The genus *Metacyclops* Kiefer, 1927 is a species-rich (50+) cosmopolitan group represented by only seven species in East and South Asia: *Metacyclops minutus minutus* (Claus, 1863) from China, *Metacyclops pectiniatus* Shen & Tai, 1964 from China and Malaysia, *Metacyclops ryukyuensis* Ishida, 1995 from Japan (Ryukyu Islands), *Metacyclops malayicus* (Kiefer, 1930) from Indonesia (Sumatra), *Metacyclops communis* (Lindberg, 1938) and *Metacyclops margaretae* (Lindberg, 1938) from India (Kiefer 1930, Lindberg 1938, Shen and Tai 1964, Lim and Fernando 1985, Ishida 1995, Dussart and Defaye 2006).

The cyclopid fauna in Vietnam is one of the least known in the Oriental region with only 26 species allocated in nine genera (*Halicyclops* Norman, 1903, *Eucyclops* Claus, 1893, *Paracyclops* Claus, 1893, *Ectocyclops* Brady, 1904, *Tropocyclops* Kiefer, 1927, *Mesocyclops* G.O. Sars, 1914, *Thermocyclops* Kiefer, 1927, *Microcyclops* Claus, 1893 and *Graeteriella* Brehm, 1926) reported so far; one-third of which belongs to the genus *Mesocyclops* (Nam et al. 2000, Tran and Ho 2009, Tran and Chang 2013). *Metacyclops* has not yet been recorded from the country. The species described herein has been collected during a zoological expedition of the Museum and Institute of Zoology PAS (Warsaw) in the Tam Đao Mts., North Vietnam, sponsored by the Polish Academy of Sciences in 1999.

## Material and methods

Specimens fixed in 5% formalin on the spot were transferred to 70% ethanol in the laboratory. Alcohol-preserved specimens were dissected in glycerine under an Olympus zoom stereomicroscope. Slide preparations were sealed with nail-polish. Light-microscopy examinations were made with Olympus BX 50 compound microscope with Nomarski optics, the pencil drawings were made with aid of a drawing tube attached to the compound microscope. Ink drawings were scanned and edited with the computer program GIMP and Inkscape.

### Abbreviations used in the paper

Enp endopodite

Exp exopodite

P1−P4 leg 1 to leg 4

MIZ Museum and Institute of Zoology Polish Academy of Sciences, Warsaw.

## Taxonomic accounts

### Order Cyclopoida Rafinesque, 1815 Family Cyclopidae Rafinesque, 1815 Genus *Metacyclops* Kiefer, 1927

#### Metacyclops amicitiae sp. n.

Taxon classificationAnimaliaCyclopoidaCyclopidae

http://zoobank.org/5AD1E073-6015-4409-93D0-306E45975CD7

##### Material examined.

**Holotype**: female dissected on two slides [MIZ 6/2015/1], Vietnam, Tam Đao, 21°45'N 105°64'E, ca. 930 m above sea level, water seeping into a shallow pit dug in gravel deposit of a creek (no name), leg. M. Hołyńska 02 Apr. 1999. **Paratypes**: six females on two slides each [MIZ 6/2015/2, MIZ 6/2015/3, MIZ 6/2015/4, MIZ 6/2015/5, MIZ 6/2015/6, MIZ6/2015/7], and two males on one slide each [MIZ 6/2015/8, MIZ 6/2015/9] from the same sample as the holotype; two females on two slides each [MIZ 6/2015/10, MIZ 6/2015/11], Tam Đao, 21°45'N 105°64'E, small rock depression filled with wet leaf litter, leg. M. Hołyńska 01 Apr. 1999.

##### Etymology.

The species is dedicated to the historical tradition of sympathy and friendship between the Poles and Hungarians. The species name *amicitiae* is the dative case of the ancient Latin word “*amicitia*,” a singular noun which means “friendship”. The gender is feminine.

Female. Length of holotype 635 µm; range and mean of body length 610–660 µm and 634 µm, respectively (n = 9). Length of prosome 413 µm (cephalothorax: 225 µm, prosomite 2: 88 µm, prosomite 3: 56 µm, prosomite 4: 44 µm). Length of urosome 222 µm. Prosome and urosome length ratio approximately 1.86. Antennule is very short. The length of antennule does not exceed the length of cephalothorax.

**Figure 1.**
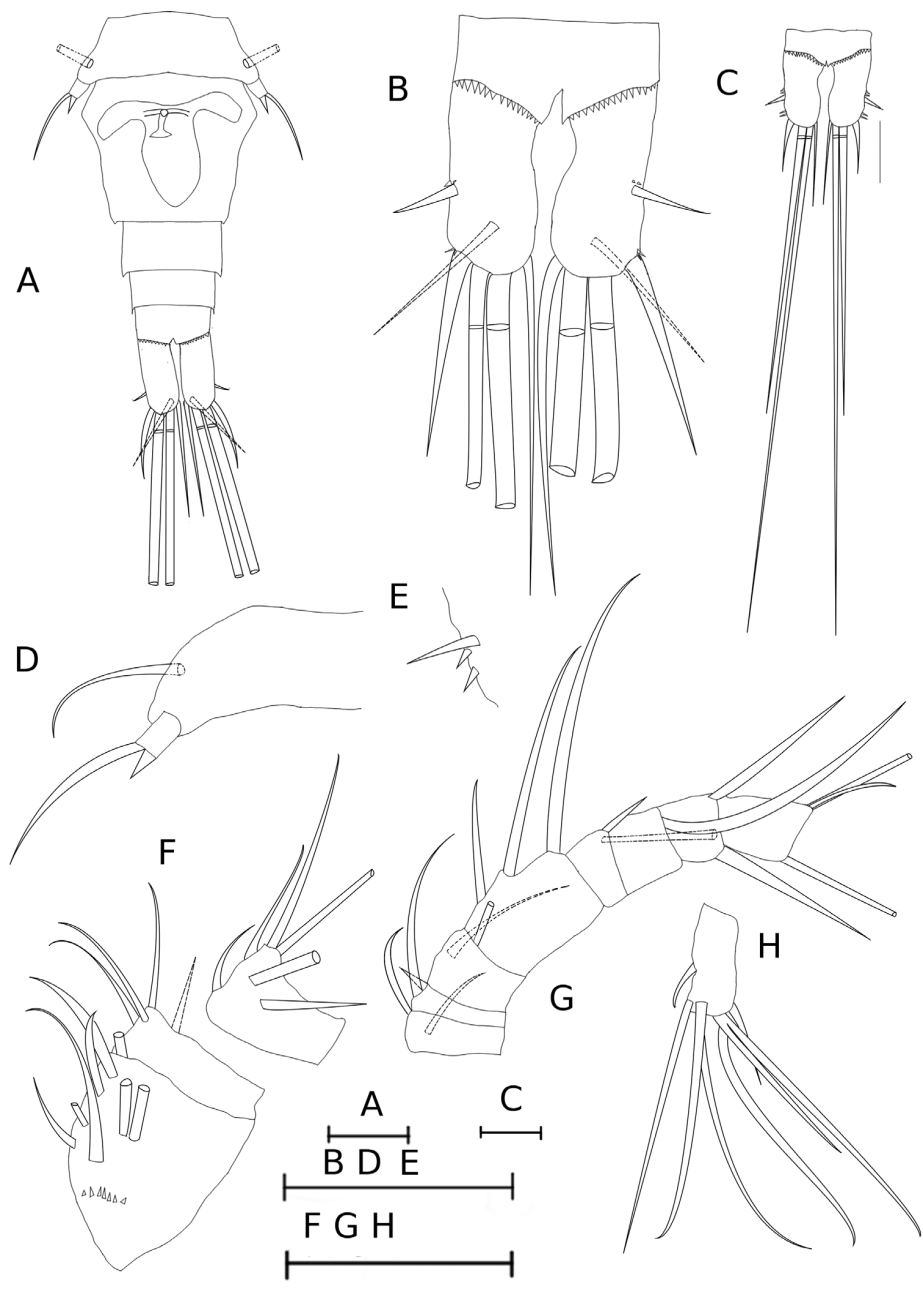
*Metacyclops amicitiae* sp. n. female. **A** urosome, ventral **B-C** caudal rami, ventral **D** leg 5 **E** leg 6 **F–H** antennule: **F** segments 1–3 **G** segments 4-11 **H** segment 12. **A–D** paratype [MIZ 6/2015/2] **F–H** holotype [MIZ 6/2015/1] **E** paratype [MIZ 6/2015/6]. Scale bars 50 µm.

Genital double-somite (Fig. 1A) about 1.21 times broader than long, no transverse ridges or hairs on the somite. Seminal receptacle “T”-shaped. Posterior margin of anal somite bearing continuous row of strong spinules.

Caudal rami (Fig. 1B, C) 2.26 times longer than wide and bearing six setae, medial margin naked. Spinules present at insertion of antero- and posterolateral caudal setae. Inner and outer terminal caudal setae with breaking plane.

Relative length of caudal setae from terminal accessory (innermost) to posterolateral (outermost) caudal setae: 1.7 (75 µm), 9.6 (422 µm), 5.5 (242 µm), 1.0 (44 µm). Dorsal caudal seta 36 µm, 0.82 times as long as posterolateral caudal seta. Setulation of caudal setae homonomous.

Antennule (Fig. 1F, G, H) 12-segmented and armed as follows: 8 (and row of spinules ventrally), 4, 6, 2, 1 + spine, 2, 3, 1 + aesthetasc, 1, 2, 2 + aesthetasc, 7 + aesthetasc.

**Figure 2.**
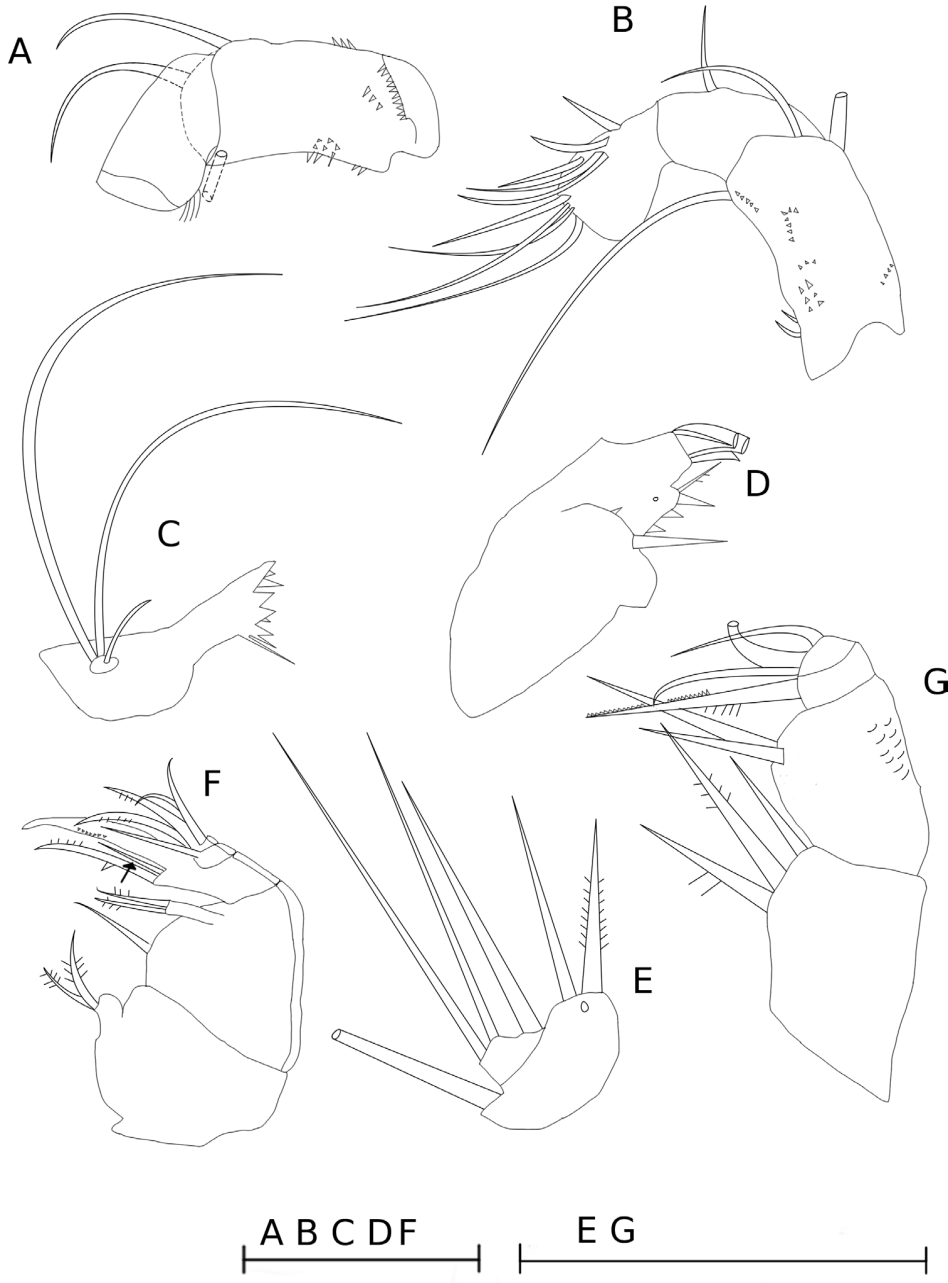
*Metacyclops amicitiae* sp. n. female, holotype. **A** antennal coxobasis and enp1, frontal **B** antennal coxobasis and enp1-2, caudal **C** mandible **D** maxillulary arthrite **E** maxillulary palp **F** maxilla, caudal **G** maxilliped, thin hairs on the frontal surface of basipodite are not shown. Scale bars 50 µm.

Antenna (Fig. 2A, B) armed with 3, 1, 9, and 7 setae on coxobasis and three-segmented endopodite, respectively. Exopodite seta long, reaching beyond enp3. Coxobasis bearing five groups of spinules on caudal surface (Fig. 2B), longer spinules present on lateral margin near base of segment, and three groups of spinules present on frontal surface (Fig. 2A).

Mandible (Fig. 2C) with reduced palp bearing two long plumose setae and short naked seta. Gnathobase with 8 teeth and dorsal seta-like element.

Maxillule (Fig. 2D, E) comprised of praecoxopodite and palp. Palp (Fig. 2E) two-segmented and bearing seven setae: three setae apically, three setae on lateral lobe (segment), and one seta proximally. Praecoxopodite has three large apical spines fused at their base, one seta on caudal surface next to base of apical spines, and seven elements on medial margin.

Maxilla (Fig. 2F) five-segmented, consisting of praecoxopodite and coxopodite (separated on caudal surface yet fused frontally), basipodite, and two-segmented endopodite. Arthrodial membrane absent between distal endopodal segment and large distal claw-like seta on caudal surface. Setal formula: 2, 3, 2, 2, 3. Short seta (Fig. 2F arrowed) inserted on caudal surface of basipodite, next to base of medial claw-like attenuation of segment.

Maxilliped (Fig. 2G) four-segmented, comprising syncoxopodite, basipodite and two-segmented endopodite. Setal formula 3, 2, 1, 3. Basipodite with thin hairs on frontal surface and one group of spinules caudally near lateral margin.

**Figure 3.**
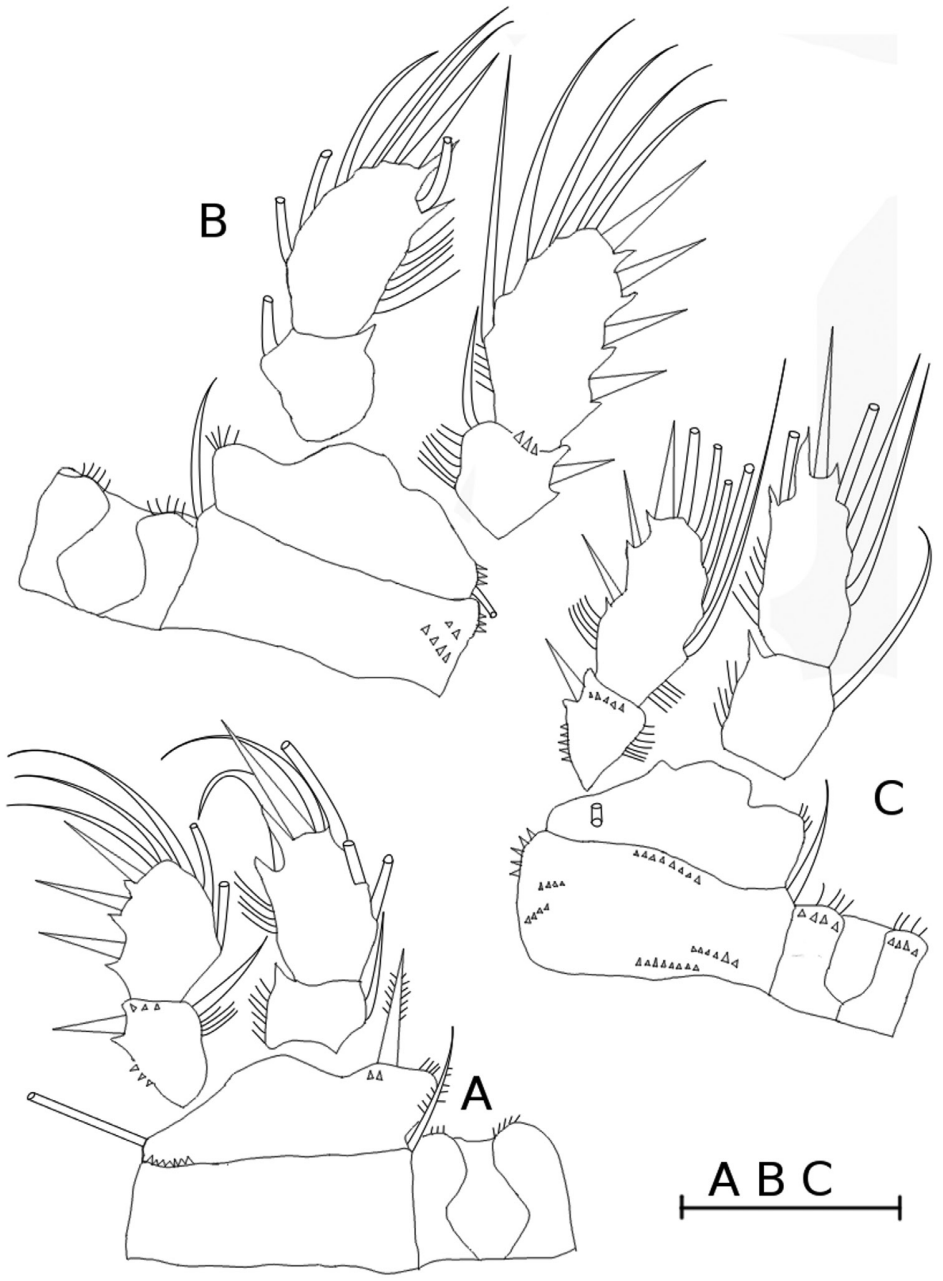
*Metacyclops amicitiae* sp. n. female, holotype. **A** leg 1, frontal **B** leg 2, caudal **C** leg 4, caudal. Scale bar 50 µm.

P1-P4 (Fig. 3) rami two-segmented, spine formula 3-4-4-3 (Table 1).

**Table 1.** Armature of the swimming legs in *Metacyclops amicitiae* sp. n. (Roman numerals indicating spines, Arabic numerals representing setae).

	Coxopodite	Basipodite	Exopodite		Endopodite	
			1	2	1	2
Leg 1	0-1	1-I	I-1	III,2,3	0-1	1,I+1,3
Leg 2	0-1	1-0	I-1	III,I+1,4	0-1	1,I+1,4
Leg 3	0-1	1-0	I-1	III,I+1,4	0-1	1,I+1,4
Leg 4	0-1	1-0	I-0	II,I+1,4	0-1	1,I,3

P1-P4: intercoxal sclerites with fine hairs on distal margin; mediodistal part of basipodite rounded and pilose. Leg 1 (Fig. 3A): coxopodite with laterodistal row of spinules. Two small spinules present next to insertion of medial spine of basipodite; spine reaching beyond distal margin of enp1. Leg 2 (Fig. 3B) coxopodite bearing two rows of spinules on caudal surface and short spinules on lateral margin. Leg 3 differing from leg 2 in size only.

Leg 4 (Fig. 3C): intercoxal sclerite with row of small spinules on caudal surface. Coxopodite bearing five rows of spinules on caudal surface and robust spinules on lateral margin. Distal segment of P4 endopodite 2.12 times as long as wide, apical spine 0.82 times as long as segment.

Leg 5 (Fig. 1A, D) with one free segment 1.6 times as long as wide, bearing one medial spine and one lateral seta. Long lateral seta inserted on laterodorsal surface of pediger 5.

Leg 6 (Fig. 1E) represented by small plate located laterodorsally in anterior fouth of genital double-somite, and bearing one seta and two subequal lateral spines; seta ca. 3 times as long as lateral spines.

Male. Length of two paratypes 537 and 539 µm. Length of prosome 334 µm (cephalothorax: 182 µm, prosomite 2: 65 µm, prosomite 3: 53 µm, prosomite 4: 34 µm). Length of urosome 203 µm. Prosome and urosome length ratio about 1.64.

**Figure 4.**
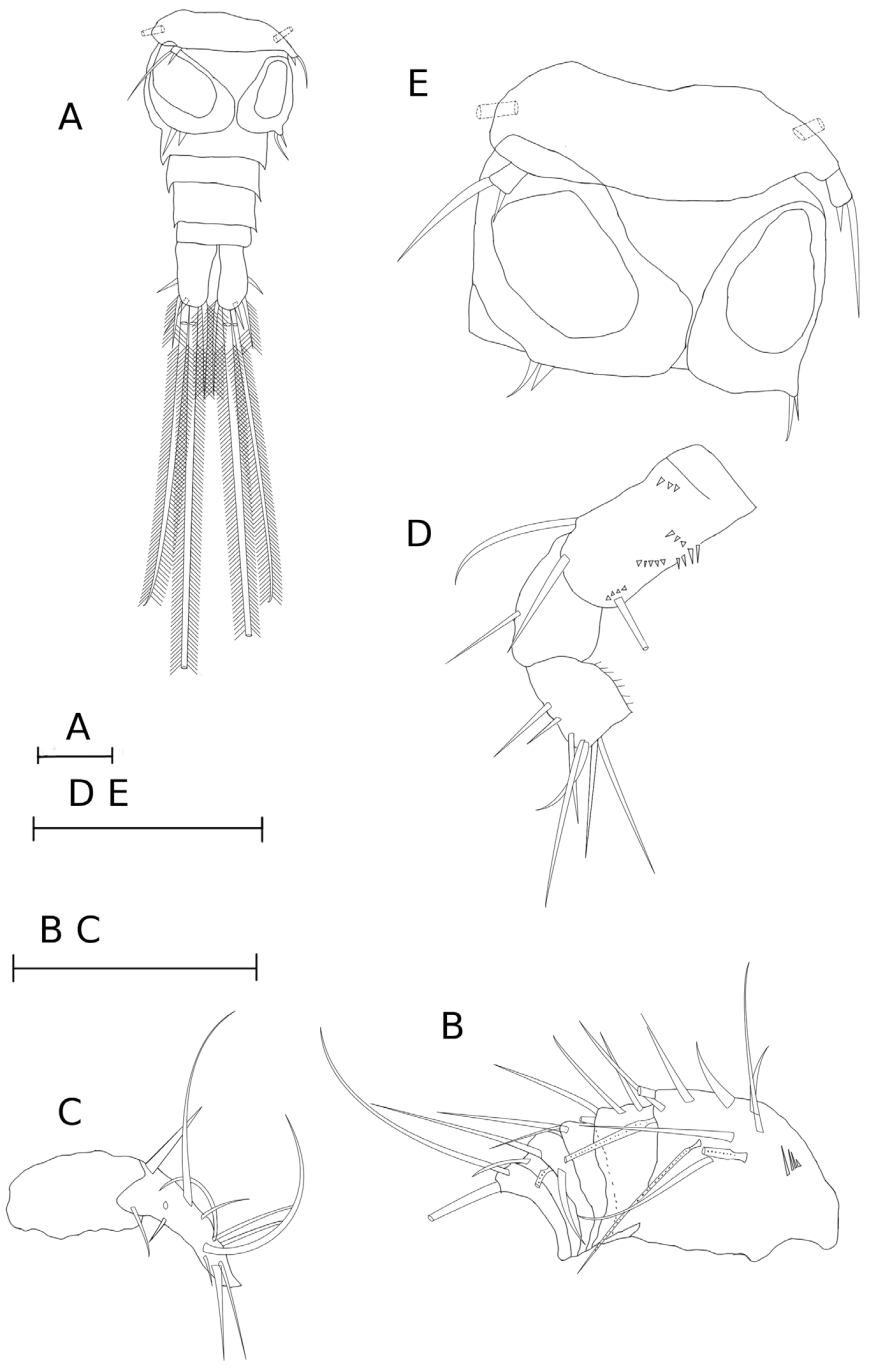
*Metacyclops amicitiae* sp. n. male. **A** urosome, ventral [MIZ 6/2015/8] **B–C** antennule [MIZ 6/2015/9]: **B** segments 1–6, ventral **C** segments 15-16, dorsal (setation is shown on segment 16 only) **D** antennal coxobasis and enp1-2, caudal [MIZ 6/2015/8] **E** pediger 5 and genital segment, ventral [MIZ 6/2015/8]. Scale bars 50 µm.

Caudal rami (Fig. 4A) 2.25 times longer than wide. Relative length of caudal setae from terminal accessory (innermost) to posterolateral (outermost) caudal setae: 1.6, 7.0, 4.2, 1.0. Dorsal caudal seta 0.88 times as long as posterolateral caudal seta. Setulation of caudal setae homonomous.

Antennule (Fig. 4B C) 16-segmented and armed as follows: 8 + 3 aesthetascs (and row of spinules ventrally), 4, 2, 2 + aesthetasc, 1, 2, 2, 2, 1 + aeathetasc+ spine, 2, 2, 2, (setation of segments 13-15 could not be verified), [4 + 1 aesthetasc and 7 + 1 aesthetasc] (one element broken one segment 16). Second endopodal segment of antenna with seven setae only (Fig. 4D). Surface ornamentation of antennal coxobasis (Fig. 4D) similar to that in female. Segmentation and setation of swimming legs, and leg 5 as in female. Distal segment of P4 endopodite 1.75 times as long as wide, apical spine 1.16 times as long as segment. Surface ornamentation of P1-P4 intercoxal sclerites similar to that in female.

P6 (Fig. 4E) bearing two elements only, lateral seta 1.6 times as long as medial spine.

##### Remarks.

**Figure 5.**
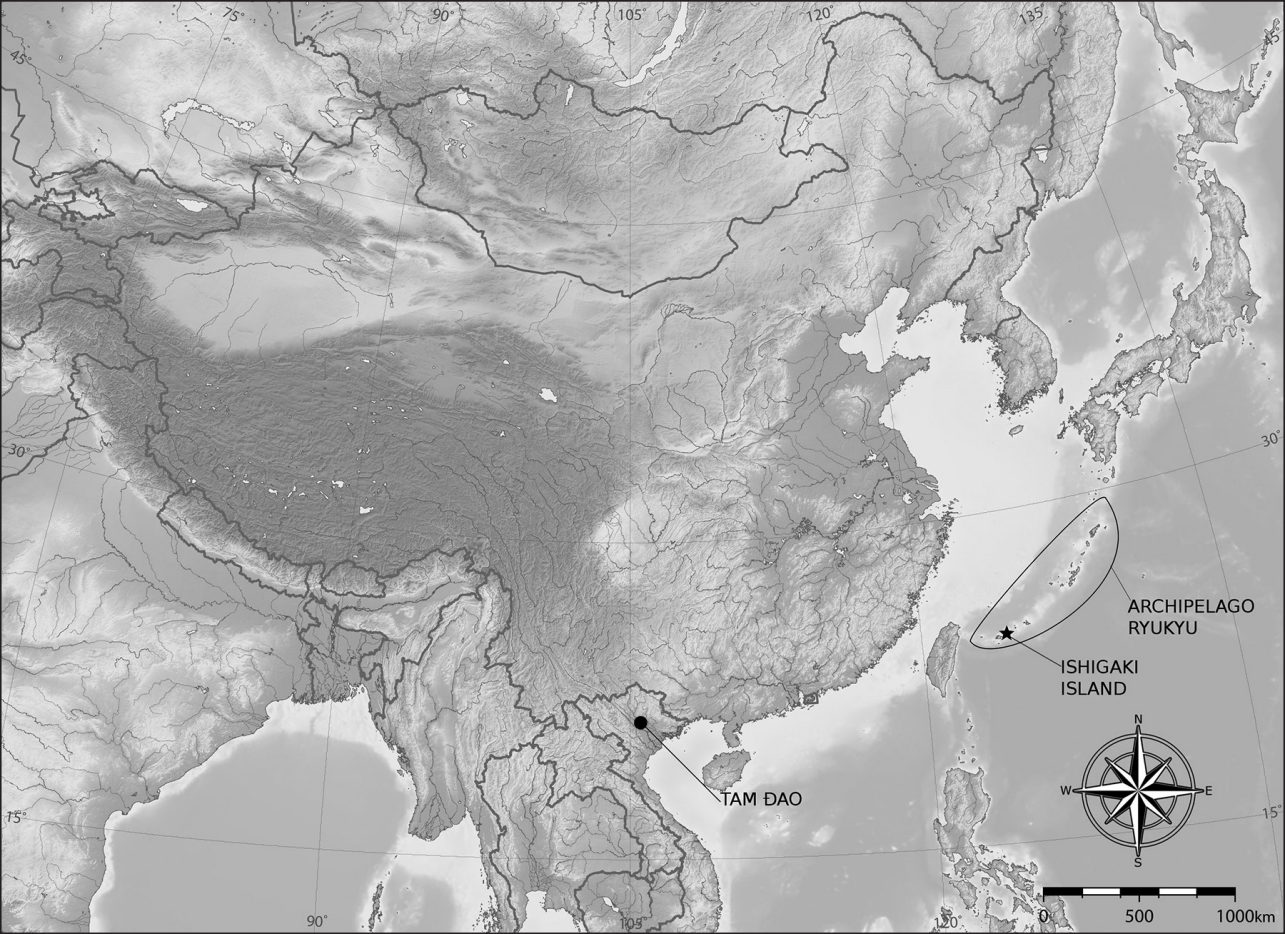
Records of *Metacyclops amicitiae* sp. n. (•) in North Vietnam and its presumed closest relative *Metacyclops ryukyuensis* (*) in Japan (Archipelago Ryukyu: Ishigaki Island).

The 12-segmented antennule in the female of *Metacyclops amicitiae* sp. n. is a very rare trait among the Old World *Metacyclops* taxa. The only other species that shows the same segmentation pattern is *Metacyclops ryukyuensis* from Ishigaki Island, Ryukyus, Japan (Fig. 5) (Ishida, 1995). However, the 12-segmented state of the antennule is not unusual among the Middle and South American taxa [*Metacyclops brauni* Herbst, 1962, *Metacyclops botosaneanui* Pesce, 1985, *Metacyclops hartmani* Herbst, 1960, *Metacyclops laticornis* (Lowndes, 1934), *Metacyclops necessarius* (Kiefer, 1926), *Metacyclops mendocinus venezolanus* Kiefer, 1956, *Metacyclops leptopus* (Kiefer, 1927), *Metacyclops leptopus mucubajiensis* Kiefer, 1956, *Metacyclops mendocinus* (Wierzejski, 1892), *Metacyclops problematicus* Dumont, 1973, *Metacyclops janstocki* Herbst, 1990, *Metacyclops hirsutus* Rocha, 1994] (see Herbst 1988, 1990; da Rocha 1994), but those New World species have two spines on the distal endopodal segment of P4, instead of the single one present in *Metacyclops amicitiae* and *Metacyclops ryukyuensis*. *Metacyclops amicitiae* sp. n. differs from *Metacyclops ryukyuensis* in the surface ornamentation of the intercoxal sclerites of the swimming legs (sclerites smooth in *Metacyclops ryukyuensis* vs. sclerites bearing distal hairs in P1-P4, and spinules on the caudal surface of P4 both in female and male of *Metacyclops amicitiae*). Other characters that also distinguish *Metacyclops amicitiae* sp. n. from *Metacyclops ryukyuensis* are: number of setae on the second endopodal segment of the female antenna (nine in the new species, eight in *Metacyclops ryukyuensis*); the size of the posterior sac of the seminal receptacle (long, approaching posterior margin of the genital double-somite in *Metacyclops amicitiae* sp. n., vs. reaching nearly the middle of the somite in *Metacyclops ryukyuensis*); P4 coxopodite with five rows of spinules on the caudal surface, robust spinules near the proximal margin of the segment in *Metacyclops amicitiae*, vs. four rows of spinules, tiny spinules near the proximal margin of the coxopodite in *Metacyclops ryukyuensis*; apical spine of P4 endopodite is 0.82 times as long as distal segment in *Metacyclops amicitiae*, vs. 0.67 times as long as distal segment in *Metacyclops ryukyuensis*; genital double-somite wider than long in *Metacyclops amicitiae*, vs. as long as wide in *Metacyclops ryukyuensis*; presence of spinules at insertion of the anterolateral caudal setae in *Metacyclops amicitiae* sp. n., vs. absence in *Metacyclops ryukyuensis*, and finally surface ornamentation of the anal sinus (naked in *Metacyclops amicitiae* sp. n., with spinules in *Metacyclops ryukyuensis*).

*Metacyclops amicitiae* sp. n. and *Metacyclops ryukyuensis* not only share several morphological characters (e.g. segmentation and setation of the antennule, mouthparts and leg morphology, relatively long terminal accessory caudal seta and small body size), but they also show similarities in habitat preference as both species seem to be related to benthic and hyporheic habitats. *Metacyclops ryukyuensis* was found in a detritus sample from a shallow stream with gravel and mud deposit (“The sample was scraped by a small hand net... from the bottom” – p. 33 in Ishida 1995). The poor information available on the geographic distribution of *Metacyclops amicitiae* sp. n. and *Metacyclops ryukyuensis* makes difficult any inference about the age of their divergence. Nonetheless, it is worth mentioning that Ryukyus Islands, which are presently isolated from Japan, China, and Taiwan by the sea (the *terra typica* of *Metacyclops rykyuensis*, Ishigaki Island, is located 240 km east of Taiwan), constituted a volcanic arc on the margin of the Asian continent (China) and separated from the Chinese mainland by the opening of Okinawa trough 1.55 million years ago (Osozawa 2013). Hence the geological history would support strong faunal relationships between subtropical Asia and the Ryukyus (see also Bănărescu 1992), and might suggest divergence of the ancestors of these *Metacyclops* species not earlier than 1.55 million years ago.

### Identification key to the South and East Asian species of *Metacyclops* (females)

[the key is based on information given in the original descriptions, except for *Metacyclops minutus*, in which a description provided by Einsle (1993) was used]

**Table:** 

1	Antennule 11-segmented	**2**
–	Antennule 12-segmented	**6**
2	Distal segment of P4 endopodite with two apical spines; spine formula 3333	***Metacyclops margaretae* (Lindberg, 1938)**
–	Distal segment of P4 endopodite with one apical spine; spine formula 3443	**3**
3	Caudal rami twice as long as wide; terminal accessory (innermost) caudal seta longer than posterolateral caudal seta (outermost terminal)	***Metacyclops malayicus* (Kiefer, 1930)**
–	Caudal rami at least three times as long as wide; terminal accessory caudal seta shorter than posterolateral caudal seta	**4**
4	Inner terminal caudal seta (longest one) ca. 1.3 times as long as outer terminal caudal seta.	***Metacyclops communis* (Lindberg, 1938)**
–	Inner terminal caudal seta >1.5 times as long as outer terminal caudal seta	5
5	P1 basipodite with medial spine	***Metacyclops pectiniatus* Shen & Tai, 1964**
	P1 basipodite lacking medial spine	***Metacyclops minutus* (Claus, 1863)**
6	P1- P4 intercoxal sclerites with hairs on distal margin and spinules present on caudal surface of P4 intercoxal sclerite	***Metacyclops amicitiae* sp. n.**
–	P1-P4 intercoxal sclerites without ornamentation	***Metacyclops ryukyuensis* Ishida, 1995**

## Supplementary Material

XML Treatment for Metacyclops amicitiae
